# Evaluating the Association Between Marijuana and Tobacco Use and the Risk of Developing Atrial Fibrillation

**DOI:** 10.7759/cureus.107662

**Published:** 2026-04-24

**Authors:** Chirag Lodha, Eric J Basile

**Affiliations:** 1 Internal Medicine, University of South Florida Morsani College of Medicine, Tampa, USA; 2 Cardiovascular Disease, University of South Florida Morsani College of Medicine, Tampa, USA

**Keywords:** atrial arrhythmia, marijuana, non-valvular atrial fibrillation, smoking tobacco, tobacco

## Abstract

Background

Marijuana and tobacco use have become increasingly prevalent, raising concerns regarding their cardiovascular effects. Atrial fibrillation (AF), a common cardiac arrhythmia associated with increased morbidity and mortality, has been linked to substance use. Nicotine in tobacco products increases sympathetic activity, heart rate, and blood pressure, predisposing individuals to arrhythmias. Similarly, tetrahydrocannabinol (THC), the primary psychoactive component of marijuana, has been associated with cardiac ion channel modulation and QT interval prolongation, although this continues to be debated. This study aimed to evaluate and compare the association between marijuana and tobacco use and the risk of developing AF.

Methods

A retrospective cohort study was conducted using the TriNetX database. Two cohorts were identified: individuals aged 18-65 with cannabis abuse diagnoses and no history of tobacco use, and individuals with documented tobacco use disorder and no documented cannabis exposure. Index events were defined as the first qualifying diagnosis. AF incidence was assessed beginning one day after the index event with no predetermined end date. Propensity score matching was performed to balance baseline demographic variables. Outcomes were evaluated using measures of association, survival analysis, and hazard ratios.

Results

After propensity score matching, the cannabis cohort included over 500,000 patients, and the tobacco cohort exceeded 1.5 million patients. AF incidence was 1.8% in the cannabis cohort compared to 2.0% in the tobacco cohort, demonstrating a statistically significant risk difference of −0.002 favoring cannabis exposure. However, the effective size of this remained small and is thus a limitation of this study. Additionally, tobacco use disorder and marijuana usage encompass two different levels of consumption, which means that the groups distinctly vary in their substance usage, which was not further quantified in this study. Mean follow-up duration was 1,435 days for the cannabis cohort and 1,696 days for the tobacco cohort. Hazard ratio analyses further supported an increased AF risk associated with tobacco use.

Conclusions

Tobacco use disorder was associated with a higher incidence of AF than with marijuana. Although this is a retrospective cohort study based on ICD-10 codes and therefore has limitations to generalizability, it suggests a potential association that should be further evaluated in randomized control trials. These findings provide important insights for clinicians and public health initiatives addressing substance-related cardiovascular risk and highlight the need for further longitudinal investigations.

## Introduction

In recent years, the consumption of both marijuana and tobacco products has emerged as a topic of significant interest regarding cardiovascular outcomes. One such cardiovascular condition that has garnered considerable attention is atrial fibrillation (AF), which is a common and clinically important cardiac arrhythmia characterized by an irregular and often abnormally rapid heart rate that can lead to serious complications [1]. AF remains a highly multifactorial disease, with risk factors including obesity, hypertension, and valvular heart disease [1]. Gaining a comprehensive understanding of the potential risks and health implications associated with marijuana and tobacco use has become increasingly critical, especially in light of the ongoing trend toward the legalization and normalization of marijuana use as well as the continued widespread prevalence of tobacco consumption. The reason why tobacco is prone to arrhythmias is multifactorial, but it is thought to be primarily due to the presence of nicotine, which is a potent stimulant that can cause an increase in heart rate and blood pressure [2]. This increase in sympathetic tone predisposes to arrhythmias via increased beta receptor stimulation. Marijuana is arrhythmogenic due to the presence of tetrahydrocannabinol (THC), which is the main psychoactive component of marijuana [3]. This can also lead to the stimulation of cardiac ion channels, causing prolonged QTc intervals as well as other types of arrhythmias, although this association is being debated [4,5]. The objective of this study is to investigate the association between marijuana and tobacco use and the risk of developing AF.

Utilizing data from the TriNetX Network, which encompasses a broad range of healthcare organizations, this research seeks to investigate the potential cardiovascular risks linked to the two substances. By identifying any significant associations, the study can inform healthcare policies and preventive measures. The ultimate goal is to investigate whether marijuana or tobacco usage poses a greater risk for AF, potentially guiding clinical practice and public health recommendations.

## Materials and methods

Study design and analytical framework

The methodological framework of this study comprised two critical steps: cohort definition and analytical setup. This was a retrospective cohort study utilizing de-identified electronic health record data from the TriNetX network. Initially, the cohorts were delineated through specific query criteria. The first cohort, termed “Cannabis AFIB,” included individuals with a diagnosis of cannabis use, aged between 18 and 65, while excluding those with documented tobacco use. However, this usage was not quantified, indicating a more heterogenous use pattern as opposed to tobacco use disorder. The second cohort, termed “Tobacco AFIB,” consisted of individuals with documented tobacco use, excluding any concurrent or prior cannabis use. This categorization ensured that the analysis could assess the risk factors associated with each substance as best as possible, as there is some degree of overlap with cannabis and nicotine usage that may not be captured by ICD-10 coding. However, as previously mentioned, true consumption patterns could not be captured and further stratified.

Index event and time window

After defining the cohorts, the analysis was configured by establishing index events and time windows. The index event denoted the initial point where cohort members met the specified criteria, serving as the baseline for outcome assessment. This, however, does not take into account those who had an extensive substance use history before the development of AF, and is a limitation of this study design, as discrete patient data could not be accessed. Subsequent outcomes, specifically incident AF, were monitored starting one day post-index event, without a predefined end date, thus including all relevant data. Any events occurring more than 20 years prior were excluded from the analysis, as this may have led to skewing of the results.

Cohort definitions

For the “Cannabis AFIB” cohort, the inclusion criteria were stringent, requiring a diagnosis of cannabis abuse between the ages of 18 and 65, as per the ICD coding system. Participants were explicitly excluded if they had any history of tobacco use. In contrast, the “Tobacco AFIB” cohort required documented tobacco use within the same age range, specifically excluding individuals with any concurrent cannabis use. The consistent application of these criteria across both groups ensured that the analysis could attribute any observed differences in AF incidence to the substance of interest. The cohort definitions were operationalized using query criteria run on the TriNetX Network. The cannabis cohort comprised over 500,000 individuals, and the tobacco cohort exceeded 1.5 million.

Propensity score matching

Propensity score matching was employed to adjust for demographic and baseline differences between cohorts, including age, sex, race/ethnicity, and comorbid conditions, which was performed by the TriNetX network analysis as part of its propensity matching program and was not able to be further accessed by the investigating team. Patients were matched 1:1 using nearest-neighbor matching with a caliper width of 0.2 standard deviations of the logit of the propensity score. Post-matching standardized differences were calculated to confirm adequate balance between groups; a standardized difference of less than 0.1 was considered indicative of adequate balance [6].

Statistical analysis

The measure of association used in this retrospective analysis was the risk ratio. Survival analysis was performed using Kaplan-Meier estimates to model the time from the index event to the first documented occurrence of AF. Survival curves were generated for each cohort, and between-group differences were assessed using the log-rank test. Hazard ratios with 95% confidence intervals were estimated using Cox proportional hazards regression. All statistical tests were two-sided, and a p-value of less than 0.05 was considered statistically significant.

## Results

Cohort characteristics

Prior to propensity score matching, the Cannabis AFIB cohort included 500,523 patients, and the Tobacco AFIB cohort included 1,559,226 patients (Table 1).

**Table 1:** Cohort sizes before and after propensity score matching.

Cohort	Description	Patients before matching	Patients after matching
Cohort 1	Cannabis AFIB	500,523	441,523
Cohort 2	Tobacco AFIB	1,559,226	441,523

Significant baseline differences were observed between the two cohorts: the cannabis cohort was younger (mean age at index 34.4 ± 12.9 vs. 45.9 ± 12.8 years; p < 0.001) and had a higher proportion of males (59.2% vs. 52.6%; p < 0.001), with large standardized differences exceeding 0.1 for all variables (Table 2).

**Table 2:** Baseline demographics before propensity score matching. The “Other/Not Reported” category includes patients whose sex was classified as other, unknown, or not documented.

Variable	Cohort 1 (Cannabis AFIB)	Cohort 2 (Tobacco AFIB)	p-value	Std Diff
Current age (Mean ± SD)	41.9 ± 13.5	52.5 ± 13.1	< 0.001	0.792
Age at index (Mean ± SD)	34.4 ± 12.9	45.9 ± 12.8	< 0.001	0.890
Female	201,370 (40.7%)	736,704 (47.3%)	< 0.001	0.132
Male	292,730 (59.2%)	819,941 (52.6%)	< 0.001	0.133
Other/Not reported	6,423 (1.3%)	2,581 (0.2%)		

After propensity score matching, 441,523 patients remained in each cohort. This was due to matching participants as closely as possible while excluding those lost to follow-up, as well as no outcome availability. Post-matching demographics demonstrated demographic balance, with standardized differences of 0.022 or less for sex and 0.014 for age at index (Table 3).

**Table 3:** Baseline demographics after propensity score matching. Cohort size = 441,523 per group.

Variable	Cohort 1 (Cannabis AFIB)	Cohort 2 (Tobacco AFIB)	p-value	Std Diff
Current age (Mean ± SD)	43.1 ± 13.6	43.1 ± 13.3	0.607	0.001
Age at index (Mean ± SD)	35.9 ± 12.8	36.0 ± 12.6	< 0.001	0.014
Female	186,445 (42.2%)	181,611 (41.1%)	< 0.001	0.022
Male	254,911 (57.7%)	259,755 (58.8%)	< 0.001	0.022
Other/Not reported	167 (<0.1%)	157 (<0.1%)		

Of note, 167 patients (<0.1%) in the Cannabis AFIB cohort and 157 patients (< 0.1%) in the Tobacco AFIB cohort had sex classified as “Other” or “Not Reported,” accounting for the discrepancy between the sum of male and female patients and the total cohort size.

Follow-up duration

After propensity score matching, the mean follow-up was 1,435.29 ± 1,456.14 days (median 1,003 days; IQR 2,186 days) for the Cannabis AFIB cohort and 1,696.46 ± 1,499.58 days (median 1,371 days; IQR 2,328 days) for the Tobacco AFIB cohort (Table 4). This demonstrates a confounding variable for analysis, as the Tobacco AFIB cohort may have had loss to follow-up within the TriNetx database or had another underlying condition that may have contributed to the group's morbidity and mortality. However, due to an inability to access discrete patient data, this remains unclear.

**Table 4:** Follow-up duration before and after propensity score matching.

Before propensity score matching				
Cohort	Mean follow-up (days)	SD	Median	IQR
Cannabis AFIB	1,492.46	1,500.09	1,058	2,275
Tobacco AFIB	1,632.39	1,365.40	1,382	2,153
After propensity score matching				
Cohort	Mean follow-up (days)	SD	Median	IQR
Cannabis AFIB	1,435.29	1,456.14	1,003	2,186
Tobacco AFIB	1,696.46	1,499.58	1,371	2,328

Atrial fibrillation incidence and risk analysis

The analysis revealed a 1.8% incidence of AF in the Cannabis AFIB cohort (7,927 of 432,412 patients) compared to 2.0% in the Tobacco AFIB cohort (8,851 of 435,161 patients) (Table 5). The risk difference was −0.002 (95% CI: −0.003 to −0.001; Z = −6.789; p < 0.001), favoring the cannabis cohort. The risk ratio was 0.901 (95% CI: 0.875-0.929), indicating a significantly lower relative risk of AF in the cannabis cohort compared to the tobacco cohort (Table 5).

**Table 5:** Risk analysis for atrial fibrillation after propensity score matching. The risk ratio compares Cannabis AFIB (Cohort 1) to Tobacco AFIB (Cohort 2); values below 1.0 indicate lower risk in the cannabis cohort.

Measures of association				
Measure	Estimate	95% CI	Z	p-value
Risk difference	−0.002	(−0.003, −0.001)	−6.789	< 0.001
Risk ratio	0.901	(0.875, 0.929)		< 0.001

Survival analysis

Kaplan-Meier analysis demonstrated that survival probability (i.e., freedom from AF) declined over time in both cohorts, with a more pronounced decline in the Tobacco AFIB cohort. At the end of the observation period, the survival probability was 88.85% for the Cannabis AFIB cohort compared with 85.29% for the Tobacco AFIB cohort. This measure indicates the time within the cohort that participants were free from AF during the average follow-up time in days in each respective cohort. This differs from the incidence of 2% in that 2% of participants developed AF, while the total sample population was free of AF 88.85% percent of the time in days since the index event in the Cannabis AFIB cohort, as opposed to the 85.29% in the Tobacco AFIB cohort. The median time to AF was not reached in either cohort, indicating that fewer than 50% of patients in each group developed AF during the study period. The log-rank test confirmed a statistically significant difference between the two survival curves (χ² = 16.845, df = 1, p < 0.001). Cox proportional hazards regression yielded a hazard ratio of 1.066 (95% CI: 1.034-1.098; χ² = 64.166; p < 0.001), indicating that tobacco users had a 6.6% higher hazard of developing AF compared with cannabis users (Table 6). However, due to a significant difference in follow-up time as well as the fact that this analysis was done using ICD-10 codes instead of direct access to patient data, these results are subject to confounding, and a randomized control trial is necessary to further examine these differences.

**Table 6:** Kaplan-Meier survival estimates, log-rank test, and Cox proportional hazards regression for time to atrial fibrillation. The hazard ratio compares Tobacco AFIB to Cannabis AFIB. “Not reached” indicates that fewer than 50% of patients developed AF during the observation period, precluding estimation of median survival.

Kaplan-Meier survival estimates					
Cohort	Patients	Patients with AF	Median survival (days)	Survival probability	SE
Cannabis AFIB	432,412	7,927	Not reached	88.85%	0.17%
Tobacco AFIB	435,161	8,851	Not reached	85.29%	0.19%
Log-Rank Test					
Statistic	Value	Degrees of freedom	p-value		
Chi-Square	16.845	1	< 0.001		
Cox proportional hazards regression					
Measure	Value	95% CI	Chi-Square	p-value	
Hazard ratio (Tobacco vs. Cannabis)	1.066	(1.034, 1.098)	64.166	< 0.001	

## Discussion

Overview of findings

This investigation explored the correlation between marijuana and tobacco usage and the subsequent risk of AF, utilizing a vast multi-center dataset from the TriNetX Network. The analysis revealed that tobacco was associated with a greater relative risk when compared to cannabis (RR: 0.901; 95% CI: 0.875-0.929; HR: 1.066; 95% CI: (1.034, 1.098), both p < 0.001). However, the effect size of this was small and limits the generalizability of the results, which is also limited by the heterogeneity of tobacco and cannabis consumption patterns and the form of their respective usages, therefore confounding these results further. However, these findings are particularly relevant in the context of increasing cannabis legalization coupled with ongoing high rates of tobacco consumption [7,8].

Tobacco use and AF risk

The elevated risk of AF among tobacco users aligns well with established pathophysiological mechanisms. Nicotine activates the sympathetic nervous system, leading to increased heart rate, heightened myocardial oxygen demand, and increased electrical instability in atrial tissues [2,9]. Moreover, chronic tobacco use has been linked with structural cardiac remodeling, atrial fibrosis, endothelial dysfunction, and persistent inflammation, all of which significantly contribute to AF development [10,11]. A meta-analysis by Aune et al. demonstrated a dose-response relationship between cigarette smoking and AF risk, with current smokers facing approximately 33% higher odds of AF compared to never-smokers [12]. The findings of the present study reinforce these known arrhythmogenic consequences and emphasize the substantial cardiovascular burden of tobacco use.

Cannabis use and cardiovascular implications

Although the association between cannabis use and AF presented a comparatively lower risk, this does not imply cardiovascular safety. THC, the primary psychoactive compound in cannabis, produces complex cardiovascular effects, including sympathetic stimulation and parasympathetic inhibition [3,13]. These actions can lead to transient tachycardia, variable blood pressure, and electrophysiological shifts such as QT interval prolongation [4,5]. Additionally, cannabis use has been linked with pro-inflammatory and pro-thrombotic conditions, potentially promoting arrhythmias [14]. A systematic review by Kariyanna et al. reported multiple case reports of cannabis-associated AF, particularly in younger patients without traditional risk factors [15]. The observed variance in AF incidence between cohorts may be attributable to different mechanisms of cardiovascular damage, as well as differences in use patterns, potency, and duration, which were not comprehensively captured in this study.

Methodological strengths

Utilizing propensity score matching enhances the study’s validity by balancing baseline demographic variables across cohorts, thus helping mitigate, but not eliminate, confounding and allowing a more accurate evaluation of the independent links between substance use and AF risk [6]. The expansive sample size sourced from the TriNetX network augments statistical power and offers broader applicability of the findings. The prolonged follow-up duration facilitated an examination of both immediate and long-term arrhythmia development, offering a detailed perspective of disease progression following substance exposure.

Limitations

Despite its strengths, this study has several limitations. The retrospective design carries risks of selection bias, residual confounding, and misclassification errors. Reliance on ICD diagnostic coding can result in inaccuracies in identifying substance use disorders and AF incidence [16]. These ICD codes also fail to capture the true substance use patterns of patients, as tobacco use disorder, as well as cannabis usage, can encompass a wide variety of heterogenous usage. Those who use tobacco may be smoking half a pack daily, while those with marijuana usage may be consuming considerably high amounts of marijuana. Due to this discrepancy in tobacco use disorder vs. marijuana usage being compared in this study, it severely limits the generalizability and results of this study. The database lacks detailed insights into frequency, exposure duration, dosages, or method of substance consumption, all of which may substantially alter cardiovascular risk. Smoked and ingested cannabis products may lead to different hemodynamic and electrophysiological outcomes [17]. Polysubstance use and other lifestyle factors such as alcohol consumption, physical activity, and socioeconomic status could not be fully controlled for in the analysis. Other diagnoses, such as alcohol use, sleep apnea, hypertension, and obesity, were also not able to be matched to any specific patient, which severely limits the generalizability of this analysis. Additionally, the varying follow-up durations between cohorts, with tobacco users having a slightly longer mean follow-up, could affect event detection, although the incident rate of AF remained higher among tobacco users despite this discrepancy. Since the study concentrated on individuals aged 18 to 65, the results may not be generalizable to older demographics, where AF is more prevalent [16]. Kaplan-Meier curves could not be exported from the TriNetX platform; therefore, we report the platform-derived survival probabilities and log-rank statistics in lieu of graphical curves. Future studies with direct access to patient-level time-to-event data should produce and publish full Kaplan-Meier curves.

Clinical implications and future directions

The clinical ramifications of these results are significant. Medical practitioners should continue to advocate for tobacco cessation to minimize cardiovascular morbidity and arrhythmia risk [17]. Although cannabis posed a lower relative risk for AF, clinicians should advise caution, especially among patients with preexisting cardiovascular conditions or susceptibilities to arrhythmias [15]. As cannabis legalization broadens, understanding its long-term cardiovascular implications becomes crucial for patient education and public health policy [8]. Future research should prioritize prospective longitudinal studies to better delineate temporal associations and causative mechanisms linking cannabis and tobacco use with AF. Explorations involving varied cannabis formulations, administration methods, and cannabinoid concentrations could help clarify the diverse cardiovascular outcomes.

## Conclusions

This study demonstrates that tobacco may be associated with a higher incidence of AF than cannabis usage, as shown by a higher relative risk (RR: 0.901 for cannabis vs. tobacco, p < 0.001). The Kaplan-Meier survival analysis demonstrated a significantly higher AF-free survival probability in the cannabis cohort (88.85%) compared with the tobacco cohort (85.29%), and the log-rank test confirmed the statistical significance of this difference (χ² = 16.845; p < 0.001). However, the mortality difference and AF-free survival may be due to the significant difference in follow-up time as well as additional confounding variables, such as dosage, frequency of consumption, and form of consumption. This study’s findings suggest that tobacco use presents a more substantial risk factor for AF compared to marijuana; however, these results are subject to several limitations within the retrospective nature of this study and a lack of access to discrete patient data.

By leveraging large datasets and employing sophisticated statistical techniques such as propensity score matching, this analysis provides further insight into how these substances affect heart health. These results underscore the necessity for continued research into the cardiovascular effects of substance use and stress the importance of targeted preventive approaches to lessen the arrhythmia burden among individuals exposed to these substances. Future research should continue to dissect these relationships, possibly exploring additional variables or longitudinal data to further elucidate long-term effects. Overall, this study contributes significant evidence to the ongoing discourse on marijuana and tobacco use, helping shape health interventions and policies aimed at mitigating the cardiovascular risks associated with these substances.
